# The invasiveness of *Hypochaeris glabra* (Asteraceae): Responses in morphological and reproductive traits for exotic populations

**DOI:** 10.1371/journal.pone.0198849

**Published:** 2018-06-14

**Authors:** Irene Martín-Forés, Belén Acosta-Gallo, Isabel Castro, José M. de Miguel, Alejandro del Pozo, Miguel A. Casado

**Affiliations:** 1 Department of Biogeography and Global Change, National Museum of Natural Sciences (BGC-MNCN), Spanish National Research Council (CSIC), Madrid, Spain; 2 Complutense University of Madrid, Department of Ecology, Madrid, Spain; 3 Autonomous University of Madrid, Department of Ecology, Madrid, Spain; 4 University of Talca, Faculty of Agricultural Sciences, Talca, Chile; Helmholtz Centre for Environmental Research - UFZ, GERMANY

## Abstract

Scientists have been interested in many topics driven by biological invasions, such as shifts in the area of distribution of plant species and rapid evolution. Invasiveness of exotic plant species depends on variations on morphological and reproductive traits potentially associated with reproductive fitness and dispersal ability, which are expected to undergo changes during the invasion process. Numerous Asteraceae are invasive and display dimorphic fruits, resulting in a bet-hedging dispersal strategy –wind-dispersed fruits *versus* animal-dispersed fruits–. We explored phenotypic differentiation in seed morphology and reproductive traits of exotic (Chilean) and native (Spanish) populations of *Hypochaeris glabra*. We collected flower heads from five Spanish and five Chilean populations along rainfall gradients in both countries. We planted seeds from the ten populations in a common garden trial within the exotic range to explore their performance depending on the country of origin (native or exotic) and the environmental conditions at population origin (precipitation and nutrient availability). We scored plant biomass, reproductive traits and fruit dimorphism patterns. We observed a combination of bet-hedging strategy together with phenotypic differentiation. Native populations relied more on bet-hedging while exotic populations always displayed greater proportion of wind-dispersed fruits than native ones. This pattern may reflect a strategy that might entail a more efficient long distance dispersal of *H*. *glabra* seeds in the exotic range, which in turn can enhance the invasiveness of this species.

## Introduction

Human actions promoting globalization around the world have enabled many plant species to overcome fundamental biogeographical barriers, reshuffling the original distribution of species [1,2]. Once in the exotic range, some of these species have become invasive, that is to say, they have overcome reproductive and dispersal barriers and have become dominant in the exotic range [3,4]. The success of the invasion process may depend on many adaptations to novel conditions over time during the range expansion of the exotic species [5].

Identifying the traits that allow exotic plant species adapting to the novel conditions and therefore enhance their invasiveness is crucial for improving prediction of species distribution shifts and control for potential invaders. Exotic plants often undergo phenotypic differentiation from their native counterparts to cope with varying environments. Phenotypic variation can be caused by two possible mechanisms: plasticity and genetic differentiation [6–8]. Complex relationships are established among the two of them. For example, on a first phase, phenotypic plasticity might facilitate the establishment of an exotic plant species in a new environment and promote its range expansion [9]. It might also spread the risk of failure on establishment by conferring acclimation to long-term unpredictability through bet-hedging strategies [10]. On a subsequent phase, selection of the optimal phenotype may occur for the exotic plant species in its exotic range [11].

When considering the role of phenotypic differentiation for invasion success, the focus should not solely be on reproductive output, but also on dispersal abilities [12,13]. It has been shown for some invasive species that the improvement in their invasiveness results from a combination of both enhanced seed output and dispersal ability which is generally related to changes in fruit morphology patterns [14,15].

Regarding seed output, exotic populations often produce more flower heads and display greater number of diaspores than their native counterparts, which in turn results in an increased propagule pressure to successfully expand the species range [16]. Exotic populations of herbaceous species usually have greater plant growth rates (i. e. above-ground biomass) in the exotic range. This can result from adaptive responses to changes in climate, water availability, soil nutrients or natural enemies (i.e. changes driven by responses to selection, which increase individual fitness in a given environment) [17–19] or from non-adaptive evolution (i.e. changes driven by stochastic events, which can be beneficial, detrimental or neutral for fitness in a given environment). As a consequence of enemy release, exotic populations potentially invest less in defense and have thus more resources left to invest in reproduction and competitive ability (EICA hypothesis; [20,21]) in the exotic range. Regarding fruit morphology of a species, it is known to be more advantageous for invasion success when species display polymorphic fruits (i. e. heterocarpy; [22]). Heterocarpic species can exploit a wider spectrum of environmental conditions and dispersal strategies for seedling establishment when colonizing new habitats [13,23].

The daisy family (Asteraceae) is overrepresented among the invasive plant species [23,24], and interestingly, most daisies exhibit fruit dimorphism. The species of study, *Hypochaeris glabra* L., is a common daisy in the Mediterranean Basin. It is native of Spanish grasslands, although it is widely naturalized in the rest of the Mediterranean-climate regions: Chile [25], California [26], Southern and Western Australia [27] and South Africa [28]. *Hypochaeris glabra* constitutes an ideal candidate to study the importance and evolution of reproductive and dispersal-related traits during invasion given its heterocarpy and its recent expansion in the Mediterranean region of central Chile (its latitudinal distribution in Chile comprises more than 2500 km, ranging from the Coquimbo Region to the Magallanes Region [25]). *Hypochaeris glabra* possesses *per se* a diversifying bet-hedging strategy due to the intrinsic characteristics of fruit dimorphism of this species. It produces dimorphic propagules that have different germination requirements, dispersing agents and dispersal pathways: heavy unbaked fruits (‘maintainer’ type [29]) with a rougher coat to allow adhesion to small mammals and light baked fruits (‘colonizer’ type [29]) with a well-developed pappus to allow wind dispersal [29–31]. Wind-dispersed fruits of this species have been shown to undergo more efficient long distance dispersal than animal-dispersed ones [29]. Thus, wind-dispersed fruits may be subjected to a high-risk strategy and strongly affected by stochastic events. Contrarily, unbaked fruits are larger and thicker, and can rely on a low-risk strategy of self-replacement to grow in the vicinity of the mother plant. Following this diversifying bet-hedging strategy, dispersal-related traits of *H*. *glabra* are expected to evolve under unpredictable environmental conditions, spreading the risk of mortality over time and enhancing long-term fitness but not the expected fitness within a generation [7,31].

Here, we aimed to explore phenotypic differentiation occurring in morphological and reproductive traits (i. e. biomass, seed output and fruit dimorphism patterns) associated with the invasive expansion of *H*. *glabra* from Spain into Chile. We collected native (Spanish) and exotic (Chilean) populations of *H*. *glabra* and grew them in the exotic range (central Chile) to explore the following question: do we observe phenotypic differentiation where plants from the exotic populations have greater plant growth, reproductive fitness and display more baked wind-dispersed fruits? We expected that plants from exotic populations would have greater biomass. Similarly, it is likely that the invasion process has been favored by plants displaying greater seed output and greater proportion of baked wind-dispersed fruits, so we expected these values to be greater for plants from exotic populations than for those from native populations.

## Materials and methods

### Study area

Our study was based in the Mediterranean grasslands of Chile and Spain. A large proportion of species from the Mediterranean Basin were introduced into Chile during the Spanish conquest of Latin America [32]. Many of these species have become naturalized mainly in the Mediterranean climatic region of central Chile although sometimes they have spread also to other climatic regions within the country. The percentage of invasive species in central Chile that belong to the daisy family is ~13.9% [32]. *Hypochaeris glabra* is one of the most abundant invasive exotic species in Chile, where it was first recorded in 1905 [33].

**Fig 1:**
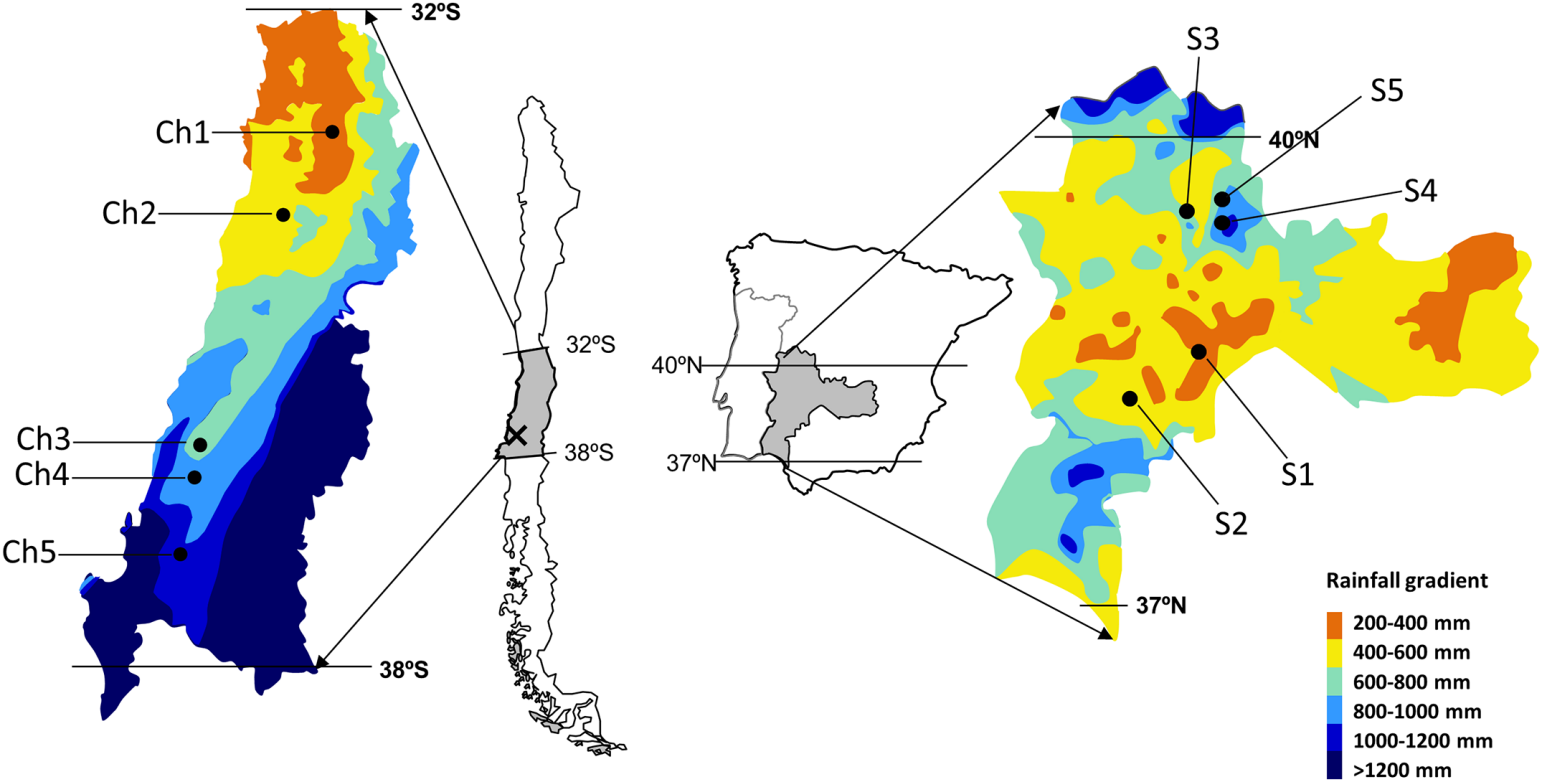
Map of the studied areas of Mediterranean grasslands in Spain and Chile, including populations sampled following a rainfall gradient (see Table 1). The location of the common garden is shown (x). This figure has been adapted from [34]. Please notice that this figure is similar but not identical to the original image, and is therefore for illustrative purposes only.

We collected *H*. *glabra* fruits from five populations in the native range in Spain and five populations in the exotic range in Chile. In Chile, populations of *H*. *glabra* were collected in the Mediterranean central region (from 32°31' to 37°00' S and 70°46’ to 72°34’ W), with mean annual precipitation between 300 and 1200 mm. In Spain, the populations were collected in the center-west of the Iberian Peninsula (from 37°51' to 40°14' N and from 4°23’ to 7°02’ W), with mean annual precipitation between 400 and 1100 mm (Fig 1; Table 1). Both regions present acid substrate, derived from igneous or metamorphic rocks, similar vegetation consisting of a continuous herbaceous layer with scattered trees (mainly *Quercus ilex* subsp. *ballota* (Desf.) Samp. in Spain and *Acacia caven* (Mol.) Mol. in Chile), and similar land use (extensive livestock grazing by sheep and cattle) [32]. The study was conducted in private lands after obtaining permission from the land owners.

**Table 1:** Geographic and climatic characteristics of the studied populations. Pop = population code; t = mean annual temperature at population origin; P = mean annual precipitation at population origin. The mean values of temperature and precipitation were calculated for the period 1970–2000. Climate data were obtained from the State Meteorological Agency (AEMET, http://www.aemet.es) and the *Atlas Climático Digital de la Península Ibérica* [35] for Spain, and from WorldClim [36] for Chile.

Country	Pop	Site	Latitude	Longitude	Altitude (m)	t (°C)	P (mm)
Chile	Ch1	Catapilco	32°35'53''S	71°18'50''W	146	16.19	352
	Ch2	Melipilla	33°49'18''S	71°18'58''W	174	17.00	412
	Ch3	Boldo	35°58'52"S	72°13'38"W	158	14.33	794
	Ch4	Quirihue	36°15'20"S	72°32'58"W	248	13.14	972
	Ch5	Yumbel	37°00'26"S	72°34'01"W	128	13.33	1168
Spain	S1	Castuera	38°46'20"N	5°34'48"W	372	16.89	468
	S2	Fuente de Canto	38°16'33"N	6°20'22"W	571	15.81	572
	S3	Madroñera	39°25'23"N	5°47'48"W	509	15.42	666
	S4	Ibor	39°32'53"N	5°22'57"W	692	14.46	859
	S5	Logrosán	39°21'28"N	5°25'04"W	453	16.17	913

### Data collection

Within the Mediterranean regions of Spain and Chile, we attempted to consider the wide environmental variability over the range of *H*. *glabra* in each country. Thus, we selected five populations in each country distributed along the rainfall gradient existing in both, Spain and Chile. Geographic coordinates, altitude and climate conditions including mean annual precipitation and mean annual temperature were recorded for each population (Table 1). In order to get information about nutrient availability for each population, five soil cores 5 cm in diameter and 12 cm in depth (excluding the aboveground biomass and litter) were sampled in 2010. The cores were pooled and homogenized, air dried and sieved through a 2-mm mesh sieve before their analysis. Soil organic matter (SOM, %), N, B, P, K and S concentrations (ppm) as well as C/N ratio were quantified using standard methods [37]. All soil samples were analyzed employing the same methodology at the Institute of Agricultural Science (Spanish National Research Council of Madrid).

In spring of 2010 (May and June in Spanish sites, and October and November in Chilean sites) we randomly collected mature flower heads from 50 individuals of *H*. *glabra* for each population; they were distributed within an area of approximately one hectare. For this study we selected unbaked seeds because they are heavier than baked ones, and their greater size favors their germination and seedling establishment [38,39]. Unbaked seeds from each population were germinated in petri dishes and then transplanted into subplots within a common garden trial located in central Chile, the exotic range. The location of the common garden was the Experimental Centre of Cauquenes-INIA, Chile (35°58’ S, 72°17’ W; 140 m a.s.l.; 14.4 °C; 748 mm of mean annual precipitation). The conditions within the common garden were controlled so there was no herbivory or competition that could affect the experiment results.

Sowing was conducted in June 2012, and the ground was previously prepared by removing surface vegetation. Ten seedlings of each population were sown in subplots of 100 x 50 cm long. The distance between plants was 20 cm. A complete randomized design was used with three replicated subplots per population. Thus, there was total of 30 subplots: 15 containing populations from Spain (5 populations*3 replicates), and 15 containing populations from Chile (5 populations*3 replicates). The total number of individuals planted was 300. However, throughout the experiment, 40% of individuals died; thus, the final count of survivors included in our study was 178 individuals. Regarding this, there were strong differences in the percentage of mortality depending on the country of origin of the populations: 55.3% ± 6.8 for Spanish populations and only 26.0% ± 10.7 for Chilean ones. For biosafety reasons, flower heads were collected from every individual after they were mature but before the infructescence opened, to ensure we captured all seeds and avoid propagules spreading.

Seed output and proportion of baked fruits were the target traits of our study because of their key role on the invasiveness of *H*. *glabra*. When plants reached ca. 50% senescence (i. e. half of the flower heads had maturated), five flower heads were randomly selected from each individual and reproductive-related traits and fruits morphology were estimated. Plants were harvested when they reached 75% senescence.

Once all plants had been harvested, we measured dry aboveground biomass (hereafter biomass) and scored reproductive and morphological traits. We counted the number of flower heads per plant. Afterwards, we counted the number of fruits present in the five flower heads that had been previously selected, separating the baked fruits from the unbaked ones. Then the average number of fruits per flower head was calculated. The proportion of baked fruits was also calculated by dividing the number of baked fruits by the total number of fruits present over five flower heads. Finally, we estimated the total seed output per plant (i. e. the total number of seeds produced per individual) by multiplying the number of flower heads per plant by the average number of fruits per flower head.

### Data analyses

All analyses were performed in R v 3.2.3 [40]. We used mixed effects models with the base stats package plus lme4 [41] to analyze phenotypic differentiation in morphological and reproductive traits of *H*. *glabra*, considering the plant individual as the unit of analysis (n = 178). Models were fitted taking into account plant growth (i. e. biomass), reproductive traits (i. e. number of flower heads per plant, average number of fruits per flower head and estimated seed output per plant) and fruit dimorphism patterns (i. e. proportion of baked fruits) as response variables. Fixed effects included the country of origin (Spain and Chile), the precipitation at population origin (as populations were selected along a rainfall gradient) and the nutrient availability at population origin (calculated as the first axis of a Principal Component Analysis (PCA) with all the soil variables, PC1). The subplot where populations were planted in the common garden was included as random effect nested within population. All the possible models including origin, precipitation and nutrient availability as predictors (as well as their interaction) were computed.

We used mixed effects models with a Gaussian error distribution for biomass, average number of fruits per flower head and estimated seed output per plant. For the number of flower heads per plant we computed mixed effects models with a Poisson family and a log-link function. Finally, for the proportion of baked fruits, we created a binomial response (number of baked fruits over five flower heads: number of unbaked fruits over five flower heads); afterwards, we computed a mixed effects model with a binomial error and a logit-link function over the binomial response.

We compared the possible models differing in the structure of fixed effects fit by maximum likelihood whether they had a Gaussian error distribution and the Laplace approximation when they had a Poisson or a binomial error distribution. We calculated the Akaike Information Criterion corrected for small sample size (AICc). We selected the best-fit models (lowest AICc presenting differences in their AICc lower than 2; [42]) with the package AICcmodavg [43]. The parsimony principle was applied on the subset of best models based on AICc and the model with a lower number of parameters was chosen for subsequent analyses [44]. Selected models were fitted by Restricted Maximum Likelihood and significant values for fixed effects were calculated with a type-III ANOVA analysis employing the package lmerTest [45]. Model validation of the best-fit model was based on visually assessing the normality of residuals. To test overdispersion we checked that the residual deviance was lower than the residual degrees of freedom [46].

We also performed mixed-effects models for reproductive traits (number of flower heads per plant, average number of fruits per flower head, and estimated seed output per plant) as well as for fruit dimorphism patterns (proportion of baked fruits) in which we entered biomass as predictor, precipitation as covariable and PC1 and subplot where populations were planted in the common garden nested within population as random effects. Additionally, we performed mixed-effects models for reproductive traits including precipitation as predictor and PC1 and subplot where populations were planted in the common garden nested within population as random effects. These models were performed by splitting the plant individuals by origin (i. e. Spanish and Chilean). Marginal r coefficients of these relationships were obtained per country of origin employing the R package MuMIn [47].

Outliers that exceeded three times the interquartile range were removed prior to analyses, which only occurred for 1.5% of cases.

## Results

**Fig 2:**
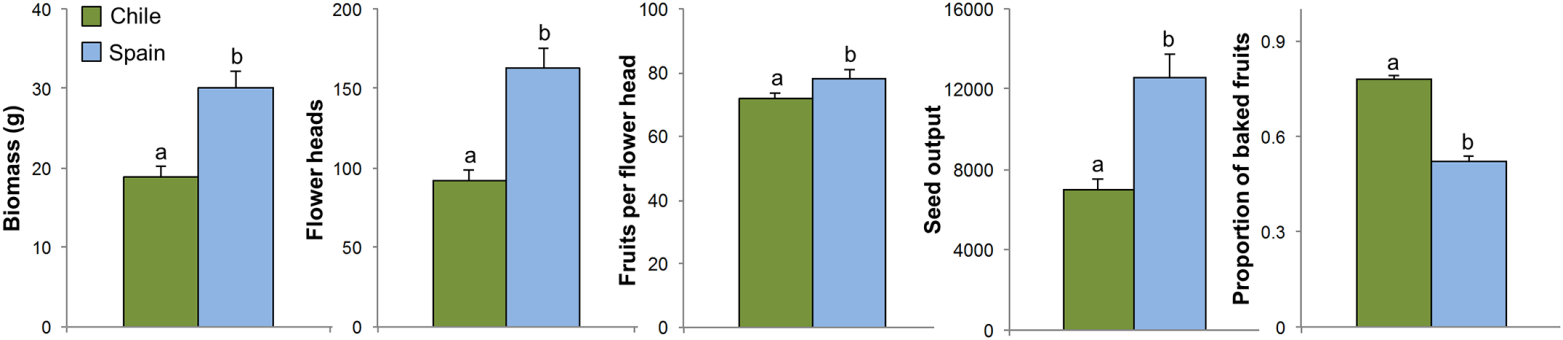
Mean values and standard errors for (a) biomass, (b) number of flower heads per plant, (c) average number of fruits per flower head, (d) estimated total seed output, and (e) proportion of baked fruits in Chilean (green bars) and Spanish (blue bars) populations. Significant differences between origins are shown with letters (a, b).

According to the generalized linear mixed-effects models, country of origin of the populations was the fixed factor that explained most of the variation of our response variables (Table 2). In the case of plant growth, country of origin had the largest effect on biomass, with significantly larger plants coming from native populations (Table 2; Fig 2). On the contrary, precipitation and nutrient availability did not have a significant effect on biomass.

**Table 2:** Effects of the country of origin (Origin) as well as annual precipitation (Precip) and nutrient availability at population origin (Nutrient) on phenotypic traits of *Hypochaeris glabra*. The table provides parameter estimates from minimal adequate (generalized) mixed effects models as well as Wald-Chisquare values and levels of significance calculated with type III Anovas for each model. First factor level: Chile; second factor level: Spain. Predictors and interactions with no significant effects are not shown in the table.

	Biomass	Flower heads	Fruits per flower head	Seed output	Proportion of baked fruits
Intercept	18.96	4.43	71.81	7158.6	1.29
	(130.90***)	(1755.55***)	(749.35***)	(63.75***)	(93***)
Origin	11.31	0.67	8.08	5330.8	-1.22
	(18.88***)	(20.17***)	(3.91*)	(14.48***)	(41.82***)
Precip		-0.24	-3.21	-1638.6	
		(10.03**)	(2.09)	(5.48*)	
Origin*Precip			19.10		
			(15.29***)		

Significance codes:

***≤0.001,

**≤0.01;

*≤0.05.

Regarding reproductive traits, all of them (i. e. number of flower heads per plant, number of fruits per flower head and seed output) were explained largely by origin and, to a lesser extent, also by precipitation. In the case of number of fruits per flower head, the interaction between origin and precipitation was also included in the best model. The values for the three reproductive traits considered were greater for the Spanish populations than for the Chilean ones (Table 2; Fig 2). Both the number of flower heads and the seed output per plant decreased with precipitation at population origin with a rate of 9.1 flower head and 647 seeds per plant each 100 mm of precipitation, respectively (S1 Fig). In the case of the number of fruits per flower head, the effect of precipitation at population origin was opposite for both countries, being positive for Spanish populations and negative for Chilean ones.

**Fig 3:**
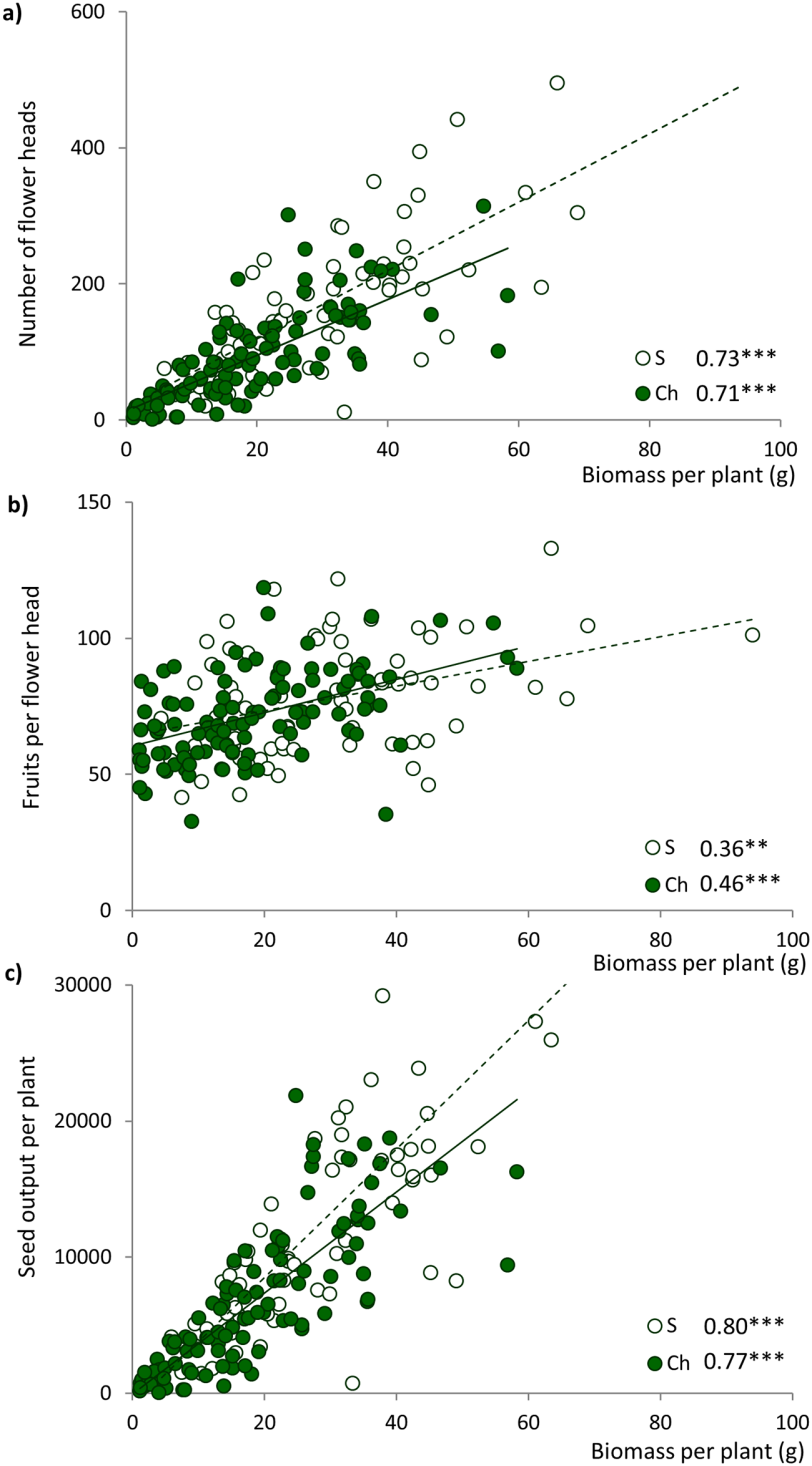
Relationships between biomass and (a) number of flower heads per plant, (b) average number of fruits per flower head and (c) estimated total seed output per plant controlling for precipitation and nutrient availability at population origin. Close circles represent Chilean populations (Ch) whereas open ones refer to Spanish ones (S). Significant relationships are shown by continuous (Chilean populations) or discontinuous (Spanish populations) lines. For each relationship, r coefficient and its significance are shown (* < 0.05; ** <0.01; *** < 0.001).

All the variables reflecting reproductive fitness were significantly and positively correlated with biomass (Fig 3a–3c), regardless of the origin of the population (number of flower heads per plant: r = 0.7; number of fruits per flower head: r = 0.4–0.5; seed output: r = 0.8).

The best model for the proportion of baked fruits only included the country of origin as a fixed factor (Table 2). Exotic populations always had a greater proportion of baked fruits than native populations (Fig 2). Neither Spanish populations nor Chilean ones showed a significant relationship between biomass and the proportion of baked fruits (r = 0.24, p > 0.1 and r = 0.04, p > 0.1, respectively).

## Discussion

**Fig 4:**
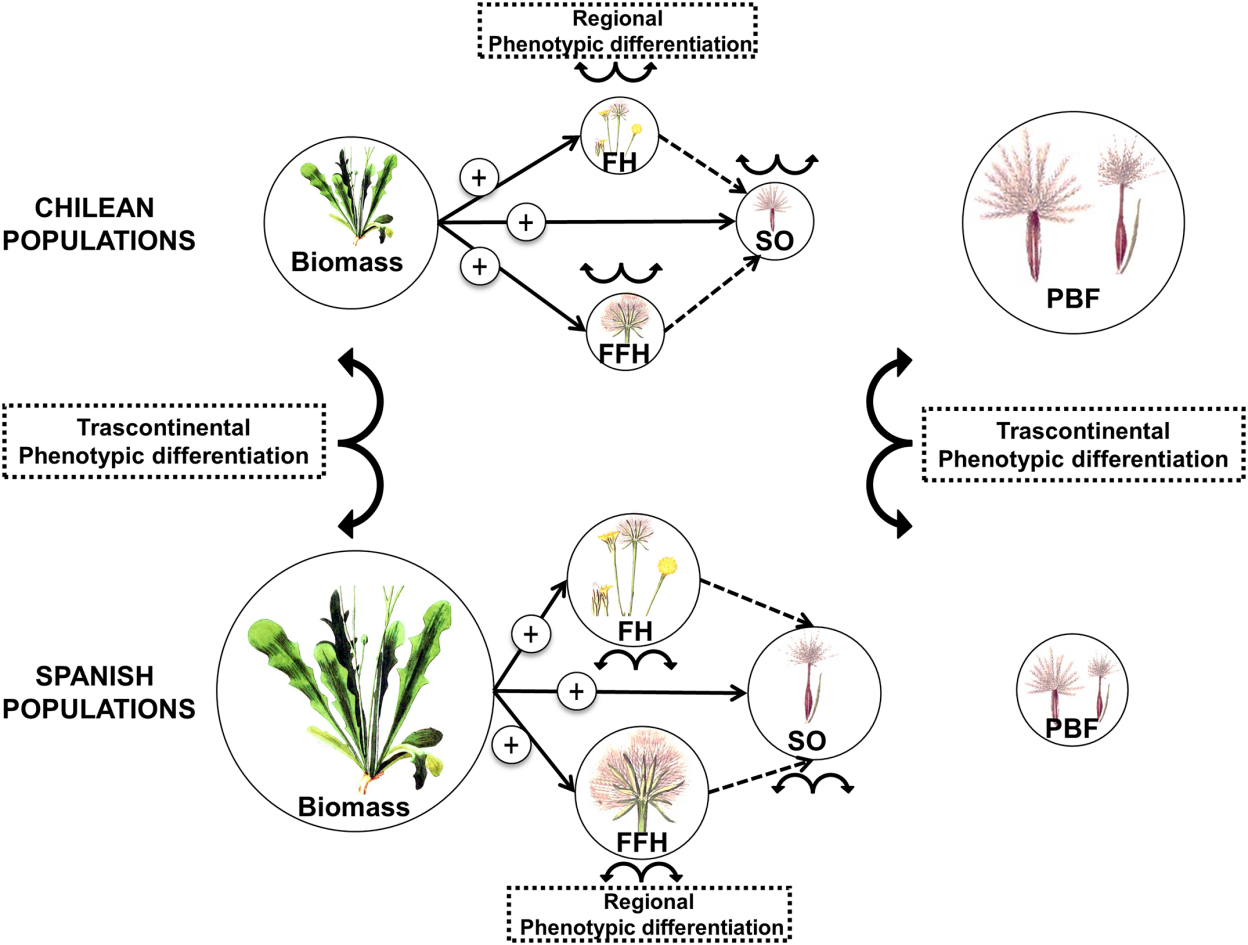
Schematic representation of the variability in plant growth (Biomass), reproductive traits (FH: number of flower heads per plant; FFH: average number of fruits per flower head and SO: estimated total seed output per plant) and fruit dimorphism patterns (PBF: proportion of baked fruits) between Chilean and Spanish populations. The size of each circle is proportional to the value of the variable. Divergent arrows represent phenotypic differences between countries (transcontinental) as well as among population within each country (regional). Black arrows (and signs) show significant relationships. Seed output was calculated as the product of FH and FFH (dashed lines). The graphic images are adaptations from J. Kops and H. C. Van Hall (1844). *Flora Batava*, vol. 8, Amsterdam (www.biolib.de).

The need to carry out comparative studies of native *versus* exotic populations of the same species in order to detect possible evolutionary change in invaders has been highlighted in the scientific literature [48]. Comparisons conducted in the exotic range are of special importance because they constitute the most direct test of determinants of invasiveness [49]. In this sense, the current study provides useful information as it checks for phenotypic differentiation in key traits for invasiveness (i. e. reproductive and dispersal-related traits) between native and exotic populations coming from a broad rainfall gradient. However, our results should be carefully interpreted with respect to adaptive evolution, taking into account that we have only included five populations per country and that we did not consider other possible drivers for phenotypic differentiation (e.g. maternal effects, prior evolutionary history, interpopulation gene flow). Additionally, the fact that we only selected unbaked achenes might have had an effect in our results that we cannot control for, although as far as we are aware no studies have noticed differences in the percentage of unbaked vs baked achenes displayed by individuals coming from one type of fruit or the other. *Hypochaeris glabra* is one of the five most frequent exotic species in Chile [32], which provides further evidence of its success in colonizing novel habitats [34]. This daisy displayed a bet-hedging strategy combined with phenotypic differentiation for the proportion of baked wind-dispersed fruits, which was significantly greater for Chilean exotic populations than for Spanish native ones. These fruit dimorphism patterns might have been determinant in order to achieve successful invasion from the native Spanish range into Chile. Plant growth and reproductive fitness also showed phenotypic differentiation at transcontinental scale (i. e. the values differed between country of origin, but they were greater for native populations than for exotic ones) as well as at regional scale (i. e. within each country, there were differences among populations associated with the annual precipitation of each population but not with the nutrient availability; Fig 4). All these results highlight the ability of *H*. *glabra* to display phenotypic changes in a short time, similar to those pointed out for other annual daisies [50,51] and exotic species subjected to novel environmental conditions [52,53]. The fact that the Spanish populations of *H*. *glabra* showed a much higher mortality rate than the Chilean ones under the novel environmental conditions of the exotic range further supports that there may have been rapid adaptive evolution during the invasion of *H*. *glabra*. Exotic populations of this species might have overcome hydric stress by evolving resistance mechanisms presumably costly that may therefore trade off against biomass and seed output. Thus, in order to successfully invade the new environment, despite considerably reduced seed output (but therefore increased survival under stress) the proportion of baked seeds increases in exotic populations.

Both exotic and native populations of *H*. *glabra* seem to display very different strategies. Exotic populations most likely rely on an expansion strategy by prioritizing the production of baked fruits as a successful way to potentially increase the distance of seed dispersal in the exotic range. In contrast, native populations of this species growing in the exotic range show a more confined strategy under the uncertainty of the novel conditions. They show greater above-ground biomass to be invested in reproductive fitness (high correlations between biomass and seed output; Fig 3) and lower proportion of baked wind-dispersed fruits. Additionally, Spanish populations show a broader range of the proportion of baked fruits produced in comparison with Chilean populations (from 1% to 70%, and from 47% to 100%, respectively) relying more on a bet-hedging mechanism to cope to the new environmental conditions.

Historical factors, such as time since the arrival of a species and its introduction pathways are determinant in its invasiveness [54]; thus, the longer a species has been in a novel environment the more chances it has to have expanded its area of distribution in the exotic range. Since the relatively recent arrival of *H*. *glabra* into Chile in 1905, this species has become widely distributed (from 30°S to 55°S latitude) and is present in many areas far beyond the Mediterranean-climate [25], indicating an unexpectedly high invasiveness. Rapid range expansion occurs when the species is highly dispersive, and a large amount of phenotypic differentiation is expected to be associated to these high dispersal rates [55].

Martín-Forés et al. [56] recently carried out a similar study for *Leontodon saxatilis* subsp. *rothii* Maire, a daisy native from Spain that is the most frequent invasive daisy in central Chile, which also has fruit dimorphism. They found that exotic populations of *L*. *saxatilis* had enhanced dispersal ability and they did not have significant differences regarding biomass and reproductive fitness in relation to the native counterparts. However, contrarily to what it would be expected for a range expansion of a successful invasive species [57], exotic populations of *H*. *glabra* showed lower biomass and reproductive fitness (i. e. number of flower heads, number of fruits per flower head and seed output), although they displayed greater proportion of wind-dispersed fruits than the native counterparts. The reduction of plant growth and reproductive fitness in exotic populations of *H*. *glabra* does not necessarily entail a reduction of the long-term fitness. In fact, the observed detriment in fitness in a single generation (or even during short periods of time) is a common tendency for species that rely on a bet-hedging strategy [7,31]. Thus, fruit dimorphism appears to be a key bet-hedging strategy for invasive species such as *H*. *glabra* along the whole introduction–naturalization–invasion continuum. The success during the first stage of invasion (i. e. establishment in the novel environment) highly increases due to multiple strategies such as different types of seeds [21]. Similarly, the number and type of propagules together with the dispersal pathway are critical factors determining the success in the species spread [13,23]. And finally, species life form and strategy, such as opportunistic dispersal by a number of dispersing agents also determines the ability of a species to become invasive [58]. The success observed for the invasive daisy *H*. *glabra* in Chile supports this idea. Considering the above mentioned, the potential long-term fitness of exotic populations of this species should not be overlooked. Future studies should assess the fitness variation between native and exotic populations of invasive species along time which provide valuable information for monitoring and decision making processes.

In summary, the origin (native or exotic) of the populations of *H*. *glabra* seems to be determinant for plant growth, fitness and main dispersing agent. Relying on a combination of bet-hedging strategies and phenotypic differentiation, exotic populations of *H*. *glabra* enhance their proportion of wind-dispersed fruits achieving more efficient long distance dispersal which potentially results in a successful invasion for this species. Plant growth and reproductive fitness of exotic populations do not keep up with the values of native populations. Rather, the *a priori* reduction of the fitness observed within a generation and the combination of both, bet-hedging and phenotypic differentiation seems to be an effective strategy for this daisy to become invasive in a broad spectrum of environments.

## Supporting information

S1 Dataset Minimal manuscript dataset. (CSV)

S1 Fig Relationship between precipitation and reproductive traits: (a) number of flower heads per plant, (b) average number of fruits per flower head and (c) estimated total seed output per plant. Close circles represent Chilean populations (Ch) whereas open ones refer to Spanish ones (S). Only significant relationships are shown by continuous (Chilean populations) or discontinuous (Spanish populations) lines.
(TIF)
