# Supplementary figures and images for: The invasiveness of Hypochaeris glabra (Asteraceae): Responses in morphological and reproductive traits for exotic populations
Source: PLoS One. 2018 Jun 14;13(6):e0198849. doi: 10.1371/journal.pone.0198849 (PMC6002075; doi:10.1371/journal.pone.0198849)

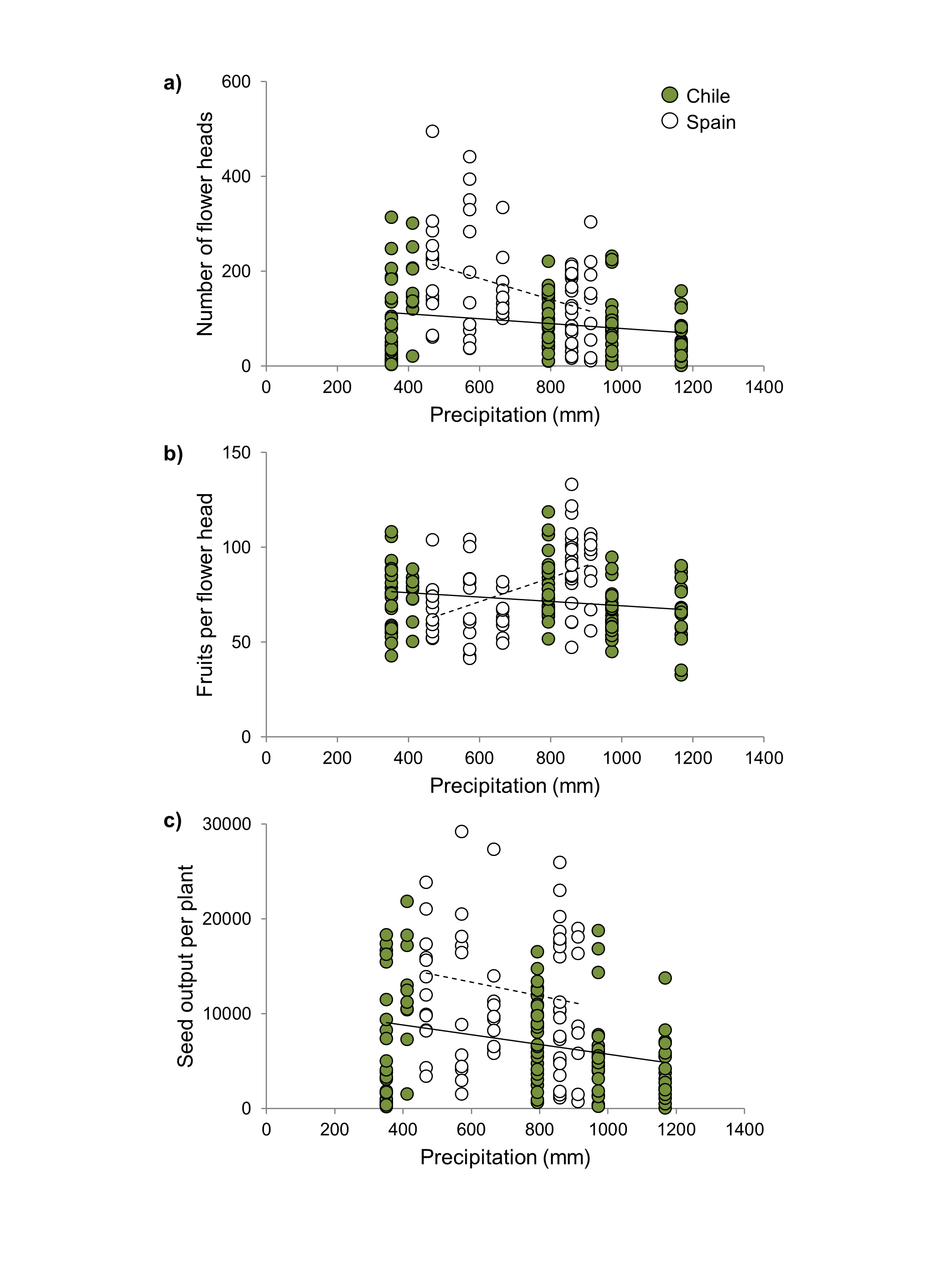

Supplement: S1 Fig — Close circles represent Chilean populations (Ch) whereas open ones refer to Spanish ones (S). Only significant relationships are shown by continuous (Chilean populations) or discontinuous (Spanish populations) lines. (TIF) [file pone.0198849.s002.tif]
